# Evaluating Glaucoma Treatment Effect on Intraocular Pressure Reduction Using Propensity Score Weighted Regression

**DOI:** 10.1038/s41598-019-52052-5

**Published:** 2019-10-29

**Authors:** Mengfei Wu, Mengling Liu, Joel S. Schuman, Yuyan Wang, Katie A. Lucy, Hiroshi Ishikawa, Gadi Wollstein

**Affiliations:** 1 Department of Ophthalmology, New York University (NYU) School of Medicine, NYU Langone Health, New York, NY USA; 2 Department of Population Health, NYU School of Medicine, NYU Langone Health, New York, NY USA; 3 Department of Environmental Medicine, NYU School of Medicine, NYU Langone Health, New York, NY USA; 4 Center of Neural Science, NYU, New York, NY USA

**Keywords:** Medical research, Optic nerve diseases, Combination drug therapy, Surgery

## Abstract

Observational studies in glaucoma patients can provide important evidence on treatment effects, especially for combination therapies which are often used in reality. But the success relies on the reduction of selection bias through methods such as propensity score (PS) weighting. The objective of this study was to assess the effects of five glaucoma treatments (medication, laser, non-laser surgery (NLS), laser + medication, and NLS + medication) on 1-year intraocular pressure (IOP) change. Data were collected from 90 glaucoma subjects who underwent a single laser, or NLS intervention, and/or took the same medication for at least 6 months, and had IOP measures before the treatment and 12-months after. Baseline IOP was significantly different across groups (p = 0.007) and this unbalance was successfully corrected by the PS weighting (p = 0.81). All groups showed statistically significant PS-weighted IOP reductions, with the largest reduction in NLS group (−6.78 mmHg). Baseline IOP significantly interacted with treatments (p = 0.03), and at high baseline IOP medication was less effective than other treatments. Our findings showed that the 1-year IOP reduction differed across treatment groups and was dependent on baseline IOP. The use of PS-weighted methods reduced treatment selection bias at baseline and allowed valid assessment of the treatment effect in an observational study.

## Introduction

Glaucoma is a leading cause of visual impairment and blindness, both in the United States and worldwide. As a chronic and complex disease, its management often requires the use of medications and/or surgery, with the primary clinical goal of lowering intraocular pressure (IOP) and ultimately halting further damage. A variety of treatment options are now available including topical and oral medications^1–5^, laser^6^, and non-laser surgery (NLS)^7^. All are designed to either decrease aqueous humor production or increase aqueous outflow. Guidance for preferred treatment is typically derived from clinical trials that are mostly designed to evaluate the effect of a single medication or surgical procedure in highly controlled settings. However, many subjects require combined treatments to halt or slow damage over the course of their disease. Thus, results from clinical trials may not reflect the complex interplay among commonly used combination regimens or may not apply to subjects who do not fit stringent trial selection criteria.

Unlike randomized controlled trials, observational studies often have pre-treatment (baseline) characteristics that are associated with treatment assignment, thereby introducing confounding effects when evaluating treatment efficacy. The propensity score (PS) method is commonly used in observational studies to reduce bias caused by different patient characteristics at baseline aiming to mimic treatment randomization^8^. PS is defined as the probability of receiving treatment assignment conditional on observed baseline covariates^9^, and combining all baseline variables into a single score is particularly convenient when there are a large number of baseline covariates^10^. Several PS methods are commonly used: PS stratification, PS matching, and PS weighting. The PS weighting method is particularly useful because all participants can be included in the study, unlike PS matching where some subjects might be excluded due to matching criteria^11^. A successful application of PS weighting can balance baseline characteristics across treatment groups to improve the estimation of treatment effects.

The purpose of this study was to determine glaucoma treatment effect on IOP reduction in real-life settings. We applied the PS weighting method for a longitudinal cohort of subjects with glaucoma that were treated with a single treatment or a combination of treatments, including medication, laser, and NLS.

## Results

One hundred twenty-two eyes (90 subjects) were included to provide 197 treatment segments of 1-year duration. The total number of segments included 33 medication, 56 laser, 33 NLS, 41 laser + medication, and 34 NLS + medication segments. Demographics are presented in Table 1. Baseline IOP differed significantly across the five treatment groups (p = 0.007), with the lowest baseline IOP in the medication group (mean ± standard deviation: 15.2 ± 5.8 mmHg), and the highest baseline IOP in the NLS group (20.3 ± 6.2 mmHg).

**Table 1:** Demographics by Treatment Group for All Segments.

	Medication (n = 33)	Laser (n = 56)	NLS (n = 33)	Laser + Medication (n = 41)	NLS + Medication (n = 34)	p value
Age (years)	64.0 (11.4)	64.4 (8.6)	63.9 (8.4)	63.0 (13.9)	65.1 (9.9)	0.90
Caucasian*	30 (88)	43 (77)	25 (76)	36 (88)	29 (85)	0.29
Female*	17 (52)	34 (61)	21 (64)	28 (68)	20 (59)	0.28
Baseline IOP (mmHg)	15.2 (5.8)	18.2 (4.7)	20.3 (6.2)	19.0 (6.0)	18.4 (5.9)	0.007**

NLS = Non-Laser Surgery; IOP = Intraocular Pressure.

*Ethnicity and gender were reported in N (%), age and baseline IOP were reported in mean (standard deviation).

**Statistically significant (ANOVA test).

**Figure 1:**
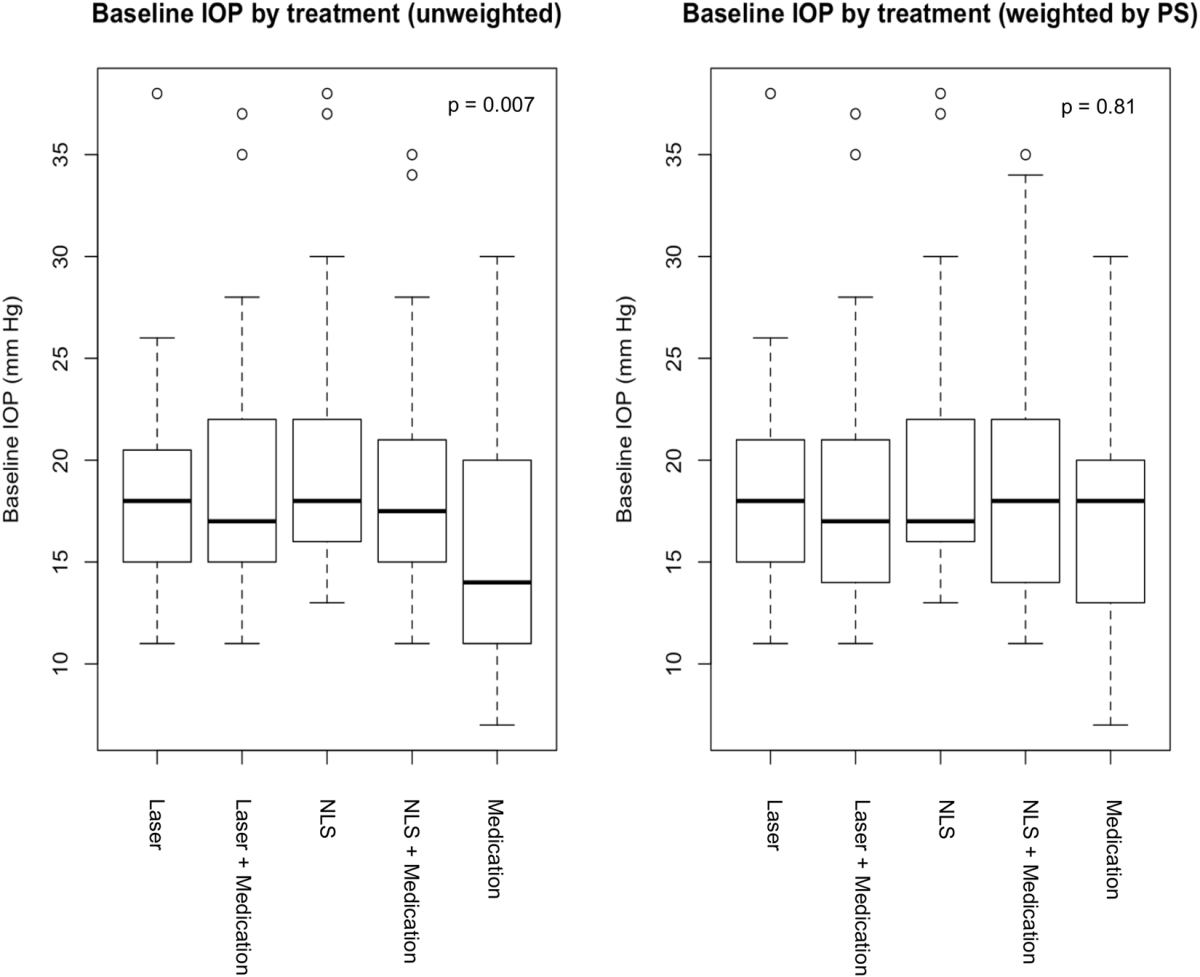
Baseline IOP by Treatment Group Before and After PS. PS = Propensity Score; NLS = Non-Laser Surgery; IOP = Intraocular Pressure.

Given the imbalance in baseline IOP across groups, we applied the generalized boosted model (GBM) for PS weighting and found that the weighted baseline IOPs were no longer different across groups (p = 0.81; Fig. 1).

We estimated the average 1-year IOP changes for all treatment groups with 95% confidence intervals (CIs) either unadjusted or adjusted by the PS weights from GBM (Table 2). Overall, the treatment groups showed statistically significant difference in their effects on IOP reduction with (p = 0.02) or without (p = 0.003) PS weighting. The NLS and NLS + medication groups showed the largest IOP decreases, and their PS-weighted estimates were −6.78 mmHg (95% CI: [−9.02, −4.54]) and −4.97 mmHg (95% CI: [−7.31, −2.62]), respectively. The medication, laser, and laser + medication groups had smaller IOP reductions. Notably, using PS weighting revealed a larger treatment effect in the medication group after correcting for the IOP imbalance at baseline. For example, the unweighted effect of medication was −1.88 mmHg (95% CI: [−3.56, −0.20]), while the PS-weighted estimate was −3.05 mmHg (95% CI: [−4.71, −1.39]). This demonstrated that the medication effect was potentially larger than it appeared to be once the baseline selection bias was adjusted.

**Table 2:** Estimated Treatment Effect by Treatment Group with 95% CI.

	1-year IOP change (mmHg) Mean (95% CI)	
	Unweighted	PS-weighted
Medication (n = 33)	−1.88 (−3.56, −0.20)	−3.05 (−4.71, −1.39)
Laser (n = 56)	−2.81 (−4.24, −1.38)	−2.79 (−4.20, −1.37)
NLS (n = 33)	−7.30 (−9.76, −4.84)	−6.78 (−9.02, −4.54)
Laser + Medication (n = 41)	−3.42 (−5.54, −1.30)	−2.82 (−4.86, −0.79)
NLS + Medication (n = 34)	−4.53 (−6.64, −2.42)	−4.97 (−7.31, −2.62)
p value	0.003*	0.02*

CI = Confidence Interval; PS = Propensity Score; NLS = Non-Laser Surgery; IOP = Intraocular Pressure.

*Statistically significant (ANOVA test).

**Figure 2:**
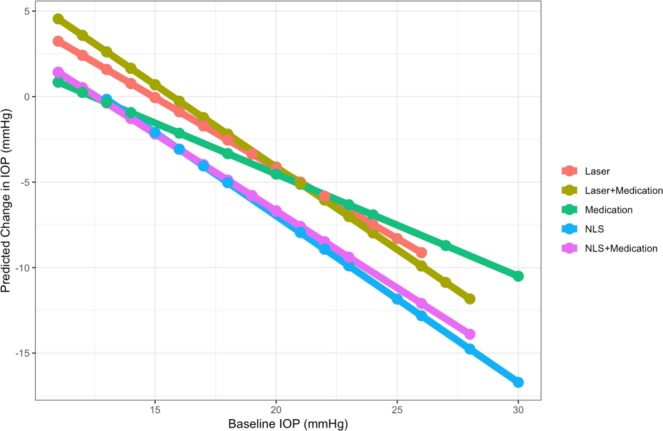
PS-weighted Predicted 1-year IOP change by Treatment Group. NLS = Non-Laser Surgery; IOP = Intraocular Pressure.

Considering that baseline IOP in the medication group was significantly lower than the other treatment groups, we further investigated the interaction between treatments and baseline IOP (Table 3) using a PS-weighted multiple linear regression model. The F-test showed that the overall interaction effect was significant (p = 0.03), mainly driven by the significant interaction between the medication group and baseline IOP (p = 0.001). Figure 2 demonstrates the results of the multiple regression model. The medication group had a significantly flatter slope than other groups due to the positive interaction with baseline IOP, indicating that medication alone became less effective than other treatments as the baseline IOP increased and was the least effective when baseline IOP >21 mmHg. The NLS and NLS + medication groups consistently showed the largest 1-year IOP reduction for patients regardless of their baseline IOPs.

**Table 3:** PS-weighted Multiple Regression on 1-year IOP Change.

Parameters (Laser as Reference)	Estimate (SE)	p value
Intercept	12.73 (1.58)	<0.001*
Medication	−6.69 (2.35)	0.006*
NLS	−1.45 (2.06)	0.48
Laser + Medication	3.57 (2.96)	0.23
NLS + Medication	−1.25 (4.05)	0.76
Baseline IOP	−0.86 (0.06)	<0.001*
Baseline IOP × Medication	0.40 (0.12)	0.001*
Baseline IOP × NLS	−0.06 (0.11)	0.57
Baseline IOP × (Laser + Medication)	−0.14 (0.16)	0.38
Baseline IOP × (NLS + Medication)	−0.01 (0.23)	0.89

PS = Propensity Score; NLS = Non-Laser Surgery; IOP = Intraocular Pressure; SE = Standard Error.

*Statistically significant.

## Discussion

Our goal was to assess the effect of medication, laser, NLS, and their combination on 1-year IOP reduction in a retrospective analysis of a longitudinal cohort of glaucoma subjects. In practice, when a clinician decides what treatment to offer, the choice is heavily influenced by the subject’s baseline characteristics and the expected outcome of treatment. Subjects with persistently high IOP at baseline might be assigned to more invasive interventions, especially if they previously failed to respond to medication. This could be a reason why baseline IOP was highest in the NLS treatment group and lowest in the medication only group. The imbalance of baseline IOP can also introduce bias in evaluating the effect of different treatments, as a bigger IOP reduction might be the result of a higher baseline IOP, not necessarily the use of a more effective treatment. Consequently, removing the imbalance in baseline characteristics is critical to achieve unbiased treatment comparison.

Our analysis successfully demonstrated that the application of PS weighting effectively reduces treatment selection bias at baseline in an observational cohort and allowed a more accurate assessment of treatment effect. Our PS-weighted results showed that treatment effects differed across five treatment groups, with NLS demonstrating a larger 1-year IOP reduction than medication and laser groups, similar to what was published by a previous meta-analysis^12^. We also demonstrated that treatment effect was dependent on baseline IOP. For example, subjects with a lower baseline IOP appeared to benefit more from medication than laser or laser + medication, while subjects with a higher baseline IOP benefitted more from treatments that included laser and NLS.

By evaluating various treatment options and aggregating interventions into medication, laser, NLS, and their combinations, we were able to estimate treatment effect and obtain results in line with current clinical knowledge and published research^13–16^. This method can be extended for further analysis considering additional treatment options, more baseline characteristics, and by accounting for factors that affect the treatment outcome such as side effects and complications. This method also has the advantage of providing strong clinical implication as it reveals the clinical reality of a longitudinal cohort, and can be used to guide treatment selection and prospective clinical studies in the future.

A post-hoc sample size calculation showed that our study was sufficiently powered, using either unweighted or PS-weighted effect estimates. Based on the unweighted estimates for treatment effects and an average between-group variance of 40 from Table 2, a sample of 145 (29 per group) would provide the study with 80% power to show difference in the mean treatment effects across groups with type I error of 5% using an ANOVA test. A more conservative power analysis using the PS-weighted estimates from Table 2 and assuming the same between-group variance showed that a sample of 200 (40 per group) would be needed to achieve 80% power.

There are a number of limitations to our study. Even though PS weighting minimized the imbalance of baseline IOP across groups, it is not a perfect substitute for a randomized clinical trial as it cannot completely remove selection bias. Also, baseline IOP was not very high in our cohort, indicating that many subjects had likely been on treatment before baseline, potentially affecting the treatment effect estimations in this study. One possible explanation to why patients with higher baseline IOPs benefited less from medication could be that within the medication group, patients with a low baseline IOP might only need a single medication to manage glaucoma; while patients with a high baseline IOP might have previously failed to respond well to a single medication and need double or triple medications. Also, some patients with higher baseline IOPs in our cohort could be referrals to our clinics after being treated elsewhere, consequently presented a greater need for surgical interventions. Furthermore, the impact of other concomitant eye surgeries, such as cataract removal, was not addressed. Prior meta-analysis has indicated that there is weak evidence that combined cataract and glaucoma surgery may result in better IOP control 1-year after surgery compared with cataract surgery alone^17^. Lastly, VF and OCT measurements, which are highly relevant in the context of treatment selection and outcome, were not considered in this study. Nevertheless, these limitations should be guarded in the context of our proof of principle study.

In conclusion, applying PS weighting to real-life clinical data reduces selection bias and allows evaluation of the IOP reduction effect of the commonly used glaucoma treatment options.

## Methods

### Subject inclusion and treatment groups

Subjects in this study came from a large, ongoing, National Institute of Health (NIH)-funded, longitudinal cohort of healthy subjects, glaucoma suspects, and subjects with glaucoma (R01 EY13178). All subjects in this cohort underwent comprehensive ophthalmic evaluation including medical history, IOP measurement, clinical examination of anterior and posterior segments, visual field (VF), and optical coherence tomography (OCT) testing. The institutional review boards and ethics committees at the New York University School of Medicine and the University of Pittsburgh approved the study. The study followed the tenets of the Declaration of Helsinki and was conducted in compliance with the Health Insurance Portability and Accountability Act (HIPAA). Informed consent was obtained from all subjects. Subjects who were diagnosed with glaucoma by their ophthalmologists and did not have confounding systemic or ocular comorbidities were qualified for the current study. Glaucoma diagnosis was based on the presence of typical structural and functional glaucomatous damage. Medication and surgical records were collected starting from the subjects’ first visit to our clinics.

Throughout follow-up, glaucoma patients could be treated with medication, laser, NLS, or a combination of medication and surgery. The following five treatment groups were considered: medication, laser, NLS, laser + medication, and NLS + medication. Medication included the use of prostaglandin analogues, beta blockers, carbonic anhydrase inhibitors, and/or alpha-adrenergic agonists. NLS included trabeculectomy, shunts/tubes, and minimally invasive glaucoma surgery. Subjects, who received a single laser or NLS intervention and/or took the same medication during a period of at least 6 months and had IOP measures before the treatment and 12-months after, were included in our study.

### 12-Month treatment segments for analysis

A treatment segment was defined as the period of time when a subject was maintained on the same treatment without adding, subtracting, or switching to a different medication or undergoing a second surgery. The first 12 months of any qualified segment were selected for analysis.

### Study outcome

IOP information was collected at each clinical visit. The last IOP measured within 3 months prior to treatment initiation was used as the baseline IOP of a treatment segment. One-year change in IOP was the outcome. If a subject did not have IOP measured at exactly the end of 12 months, two IOP measurements immediately before and after the end of one year were used to impute the value by linear interpolation.

### Statistical analysis

For continuous variables, the mean and standard deviation (SD) were reported and the analysis of variance (ANOVA) test was used to compare baseline covariates across the five treatment groups. For categorical variables, counts and proportions were reported and the chi-squared test was used to compare covariates across groups.

Generalized boosted model (GBM)^18^, a machine learning method, was used to calculate PS weights. Previous studies have shown that machine learning methods generally outperform traditional multinomial logistic models by achieving better balance among treatment groups with regards to baseline characteristics and producing more stable PS weights^19^. This is especially the case when there are more than two treatment groups, as in our study. A detailed description of the PS and GBM methods is provided in the supplementary information.

GBM analysis was performed using the Toolkit for Weighting and Analysis of Nonequivalent Groups (TWANG) R-package^20^. PS weights for treatment assignment were calculated based on each individual’s baseline IOP, age, gender, and ethnicity. To assess the performance of PS weighting in removing selection bias, a balance analysis was then conducted for baseline characteristics before and after the implementation of PS weighting^21^. Treatment effect on the 1-year IOP change before and after PS weighting was estimated with 95% CIs and compared across treatment groups using the ANOVA test.

Given that baseline IOP was unbalanced across treatment groups, a PS-weighted multiple regression model was further built to evaluate the interaction effect of baseline IOP and treatments on the 1-year IOP change. The laser group served as the reference for treatment comparison. The F-test was used to assess whether the interaction effect was significant. The estimated 1-year IOP change was plotted to demonstrate the treatment effect along the range of baseline IOPs. For all analyses, p < 0.05 was considered statistically significant.

## Supplementary information

Supplementary Information

## Data Availability

The data analyzed in this study are available from the corresponding author on reasonable request.
