# Supplementary material for: Evaluating Glaucoma Treatment Effect on Intraocular Pressure Reduction Using Propensity Score Weighted Regression
Source: Sci Rep. 2019 Oct 29;9:15496. doi: 10.1038/s41598-019-52052-5 (PMC6820863; doi:10.1038/s41598-019-52052-5)
Supplement: Supplementary file 1 — Supplementary Information [file 41598_2019_52052_MOESM1_ESM.docx]

Evaluating Glaucoma Treatment Effect on Intraocular Pressure Reduction Using Propensity Score Weighted Regression

Supplementary Information

Mengfei Wu, Mengling Liu, Joel S. Schuman, Yuyan Wang, Katie A. Lucy, Hiroshi Ishikawa & Gadi Wollstein

Supplementary Information

The propensity score (PS) is defined as the probability of treatment assignment conditional on baseline covariates. In the case of a simple, two-group study, let $Z_{i}$ be an indicator variable denoting whether or not the $ith$ subject received the treatment, and let $X_{i}$ denote a baseline covariates for the $ith$ subject. The PS $e_{i}$ for the $ith$ subject is $e_{i}=\Pr\left( Z_{i}=1 | X_{i} \right)$. Therefore, $e_{i}$ is a pre-specified value and independent of $X_{i}$ for randomized trials, but $e_{i}$ usually depends on $X_{i}$ in observational studies. PS weights are defined as $w_{i}=\frac{1}{e_{i}}$ for the treatment group, and $w_{i}=\frac{1}{{1-e}_{i}}$ for the control group. PS weights can be used to calculate causal effects for treatments such as average treatment effect (ATE). The ATE of treatment A to treatment B is the effect difference in the expected value of the outcome had the entire population received treatment A versus had the entire population received treatment B. A successful application of PS weighting can achieve good balance of baseline characteristics between treatment groups in observational studies and reduce bias in estimating ATE.

Generalized boosted model (GBM) is a frequently used machine learning method for calculating PS weights, and consists of many multiple regression trees iteratively to capture complex and nonlinear relationships between treatment assignments and baseline covariates. At each iteration, a new regression tree is added to provide the best fit to the residuals of the model from the previous iteration until the model is sufficiently flexible to fit the data. This iterative estimation procedure can be tuned to find the best balance of baseline characteristics among treatment groups. As a result, it creates a synthetic sample in which the distribution of the measured baseline covariate is independent of treatment assignment. PS weights can be easily incorporated into many statistical models such as regression, analysis of variance (ANOVA), and support vector machine for further analysis.

An outline of using GBM to estimate the ATEs is as below. First, dummy indicators are created for each of the treatment groups, e.g., receiving non-laser surgery (NLS) vs. not receiving NLS. Then, a GBM is fitted for each dummy treatment indicator to obtain the estimated PS for the given treatment (e.g., the probability of receiving NLS). Finally, the estimated PSs from each GBM are used to compute the ATE weights. To assess the success of PS weighting in removing the selection bias, a balance analysis is conducted for baseline characteristics before and after the implementation of PS weighting. Two common balance metrics are used to assess how well propensity score weights balanced the treatment groups: standardized effect size (ES), and Kolmogorov-Smirnov (KS) statistic. ES shows the pairwise mean difference of a baseline covariate between treatment groups, divided by the pooled standard deviation. ES less than 0.20 is generally considered a sign of good balance. KS statistic shows how similar the pairwise empirical cumulative distribution functions of the baseline covariate between treatment groups. The rule of thumb for good balance is a p value less than 0.05 for KS.
